# Effects of mulberry leaf silage on antioxidant and immunomodulatory activity and rumen bacterial community of lambs

**DOI:** 10.1186/s12866-021-02311-1

**Published:** 2021-09-20

**Authors:** Bing Wang, Hailing Luo

**Affiliations:** State Key Laboratory of Animal Nutrition, College of Animal Science and Technology, China Agricultural University, Beijing, 100193 People’s Republic of China

**Keywords:** Antioxidant activity, Bioactivity, *Morus alba*, Microbiome, Rumen

## Abstract

**Background:**

Rumen is a natural fermentation system and the microorganisms inside can effectively utilize plant bioresource and interact with host metabolism. Here, analysis of rumen microbiome, together with animal performance and serum metabolism in a lamb model were performed to identify the potential use of mulberry leaf silage (MS) to replace alfalfa silage (AS) as a new functional feed resource and to mining the novel specific mulberry leaf associated rumen bacteria interact with host metabolism.

**Results:**

The lambs fed with MS diet showed improved antioxidant capacity and immune function compared to those fed AS diet. The MS diet significantly altered rumen microbiota α- and β-diversity and taxonomic composition. Microbial analysis revealed that *Bifidobacterium, Lactobacillus* and *Schwartzia* were enhanced, and *Ruminococcaceae UCG-010* and *Lachnospiraceae_XPB1014_group* were down-regulated in the rumen of MS group. A strong association was also found between these rumen microbial taxa and host antioxidant and immunomodulatory capacity.

**Conclusion:**

These findings indicated that mulberry leaf silage can be a high-quality feed source or bioactive pharmaceutical that is responsible for ruminant’s health benefits. The modified rumen microbial community by mulberry leaf silage were associated with the enhanced antioxidant capacity and immunomodulatory of lambs.

**Supplementary Information:**

The online version contains supplementary material available at 10.1186/s12866-021-02311-1.

## Background

Mulberry (*Morus alba*) leaf, belonging to *Moraceous* plant, is commonly used as a Chinese traditional medicine for human beings and the sole food source for silkworms. It has a long history of cultivation throughout the world [1, 2]. The mulberry leaf is a remarkable biomass production in China as its field area and production has been estimated to be 700,000 ha and approximately 1.82 × 10^7^ t, respectively [3].

Mulberry leaf also has highly palatable and digestible (70–90%) macronutrients to herbivorous animals, especially for its relative high protein content (15–28%) and good essential amino acid profiles, which are similar to those of alfalfa hay [4, 5]. Meanwhile, mulberry leaf has antioxidant, antibacterial, and immune-enhancing properties due to its abundant bioactive phytochemicals, such as polysaccharides, flavonoids, phenolic acid, and alkaloids, and thus can be used as an alternative “green” additive in properly replacing probiotics in improving immune function and preventing disease occurring [6–8]. Thus, mulberry leaf can be used as a functional feed source or feed supplement in the diets of ruminants and monogastric animal not only because of their abundant resources and exceptional nutritional value but with important biological activity [8, 9].

However, till now, mulberry leaves have not been fully utilized as animal feed source due to its high moisture content, but ensiling is an efficient method to solve this problem [10]. It has been confirmed that the fermented well silage has more palatable features for animal preference [11, 12]. Furthermore, the mulberry leaves after ensiling could hold robust bioactive properties such as antioxidant capacity and keep the total flavonoid content and even improve their bioactive properties by additives [13]. Several recent studies found that high-quality mulberry leaf silage can be achieved by additives [1, 10, 13], but did not refer to the utilization of mulberry leaf silage. Rumen is a main site for the degradation of dietary nutrients in ruminants. The rumen microbiome is emerging as a cross bridge interacted with dietary utilization, host metabolism and phenotype changes, as shown by the association between rumen microbiome and the host metabolism [14–16].

Our questions focused on that mulberry leaf silage may have beneficial roles in the antioxidant activity and immunomodulatory of lambs. Here, this study was to assess the mulberry leaf silage that could be a high-quality feed source with important biological activity and biofeed resources for ruminants, and then to mining the potentially associated rumen microbiome accounting for its bioactive function.

## Results

### Growth and slaughter performance

As shown in Table 1, DM intake, average daily gain (ADG), body weight (BW), and the ratio between DM intake and ADG were similar between the two diets. The slaughter BW, carcass weight, and dressing percentage were similar between the two groups. The weight of head and spleen in alfalfa silage diet (AS) group lambs were greater than those in mulberry leaf silage diet (MS) group (*P* < 0.05). The weight of kidney in AS group lambs had a tendency to be lower than that in MS group (*P* = 0.053).

**Table 1:** Effect of alfalfa silage (AS) and mulberry leaf silage (MS) based diets on growth performance, organ index, and meat quality characteristics in lambs

Items	Treatments		SEM	*P*-value
	AS	MS		
Growth performance				
DMI, g/d	0.62	0.61	0.007	0.67
0d BW,	15.2	15.3	0.44	0.90
80d BW, kg	21.2	22.0	0.36	0.17
ADG, g/d	97.7	96.0	7.85	0.87
DMI/ADG, g/g	6.34	6.39	0.378	0.92
Organ index				
Carcass weight, kg	10.5	10.7	0.23	0.57
Dressing percentage, %	49.5	48.5	0.44	0.19
Head weight, kg	1.55	1.37	0.042	0.03
Hooves weight, kg	0.48	0.48	0.018	1.00
Pelage weight, kg	1.83	1.92	0.072	0.45
Heart, kg	89.8	84.4	3.99	0.38
Liver, kg	318.6	338.5	21.08	0.53
Spleen, kg	32.5	29.2	0.72	0.02
Lung, kg	199.4	197.8	6.30	0.87
Kidney, kg	30.1	34.5	1.22	0.05
Tail lipid, kg	720.0	835.6	54.09	0.19
Kidney lipid, kg	38.4	44.5	4.85	0.41
Meat quality				
GR, mm	12.9	13.4	0.56	0.55
pH 45 min	6.83	6.70	0.075	0.27
a* 45 min	6.27	6.81	0.394	0.38
b* 45 min	6.68	6.84	0.748	0.89
c* 45 min	9.48	9.80	0.743	0.77
H* 45 min	46.4	44.3	2.27	0.53
L* 45 min	34.1	32.2	0.67	0.10
pH 24 h	5.66	5.55	0.074	0.32
a* 24 h	8.60	9.18	0.520	0.46
b* 24 h	10.7	11.1	0.68	0.71
c* 24 h	13.8	14.4	0.80	0.59
H* 24 h	51.4	49.9	1.17	0.42
L* 24 h	41.2	41.0	0.62	0.78

*DMI* dry matter intake, *BW* body weight, *ADG* average daily gain, *GR* the depth of muscle and fat tissue from the surface of the carcass to the lateral surface of the 12th rib 110 mm from the midline, a* redness, b* yellowness, c* Chroma, H* Hue angle, L* lightness, *SEM* standard error of means

### Meat quality characteristics

Meat quality characteristics were similar between the two diets, as shown in Table 1. Compared with the AS diet group, the lightness (L*) value at 45 min had a tendency to be lower in the MS diet group compare to AS group (*P* = 0.10).

### Nutrients metabolism, antioxidant activity, and immune response

Overall, serum lipid metabolite levels including the concentrations of cholesterol, triacylglycerol, and the high density lipoprotein cholesterol (HDL) and low density lipoprotein cholesterol were un-affected by the MS diet (Table 2). The serum blood urea nitrogen (BUN) concentration was greater in the MS group than in the AS group (*P* < 0.01) but not for the serum albumin and globulin. As shown in Table 2, the activity of serum catalase (CAT), glutathione peroxidase (GSH-PX), superoxide dismutase (SOD), and total antioxidant capacity (T-AOC) were significantly greater in the MS group than in the AS group (*P* < 0.05). The content of malondialdehyde (MDA) was significantly lower in the MS group than in the AS group (*P* < 0.05). Compared to the AS group, the serum interferon-γ (IFN-γ) concentration was significantly higher in the MS group (*P* < 0.05), and the concentrations of serum IL-1β and IL-6 was significantly lower in the MS group (*P* < 0.05). No significant difference was found for the tumor necrosis factor-α (TNF-α) and IL-2 concentrations between the two groups.

**Table 2:** Effect of alfalfa silage (AS) and mulberry leaf silage (MS) based diets on serum biochemical, antioxidant, and immune characteristics in lambs

Items	Treatments		SEM	*P*-value
	AS	MS		
Total protein, g/L	63.0	64.2	1.19	0.49
Albumin, g/L	28.9	28.7	0.42	0.78
Globulin, g/L	34.1	35.5	0.83	0.28
BUN, mmol/L	6.64	7.00	0.061	0.01
Glucose, mmol/L	5.06	5.34	0.275	0.50
Total cholesterol, mmol/L	1.47	1.57	0.143	0.63
Triglyceride, mmol/L	0.60	0.67	0.059	0.42
HDL, mmol/L	0.54	0.57	0.048	0.63
LDL, mmol/L	0.81	0.86	0.084	0.64
IgA, g/L	0.64	0.61	0.018	0.37
IgG, g/L	17.2	17.6	0.17	0.19
IgM, g/L	1.13	1.12	0.057	0.90
CAT, U/ml	12.2	12.7	0.09	0.02
GSH-PX, U/ml	952.4	979.4	6.79	0.04
MDA, nmol/ml	4.96	4.54	0.098	0.03
SOD, U/ml	94.1	97.4	0.82	0.03
T-AOC, U/ml	9.99	10.57	0.141	0.03
TNF-α, pg/ml	44.9	45.9	0.70	0.34
IFN-γ, pg/ml	154.5	157.7	0.67	0.02
IL-1β, pg/mL	18.3	16.5	0.53	0.04
IL-2, pg/mL	157.2	163.0	2.41	0.12
IL-6, pg/mL	44.6	37.4	1.90	0.02

*BUN* blood urea nitrogen, *HDL* high-density lipoprotein cholesterol, *LDL* low-density lipoprotein cholesterol, *IgA* immunoglobin A, *IgG* immunoglobin G, *IgM* immunoglobin M, *CAT* catalase, *GSH-PX* glutathione peroxidase, *MDA* malondialdehyde, *SOD* superoxide dismutase, *T-AOC* total antioxidant capacity, *TNF* tumor necrosis factor, *IFN* interferon, *IL* interleukin, *SEM* standard error of means

### Fermentation characteristics

As shown in Table 3, rumen pH value and the concentrations of ammonia-N and total volatile fatty acids were similar between the AS and MS groups. The molar proportion of the isobutyrate had a tendency to be lower in the MS group than in the AS group (*P* = 0.09).

**Table 3:** Effect of alfalfa silage (AS) and mulberry leaf silage (MS) based diets on rumen fermentation characteristics in lambs

Items	Treatments		SEM	*P*-value
	AS	MS		
pH	5.88	5.64	0.231	0.42
Ammonia-N, mg/100 mL	24.1	31.3	5.46	0.40
Total VFA, mmol/L	110.6	128.2	18.42	0.54
Molar proportion, %				
Acetate	64.8	62.0	2.42	0.44
Propionate	19.5	24.3	2.87	0.29
Butyrate	12.2	10.7	1.31	0.45
Valerate	1.06	1.27	0.135	0.33
Isobutyrate	0.85	0.49	0.125	0.09
Isovalerate	1.51	1.29	0.330	0.66
Acetate:Propionate	3.35	3.34	0.733	0.99

*VFA* volatile fatty acids, *SEM* standard error of means

### Change in ruminal bacterial communities

**Fig. 1:**
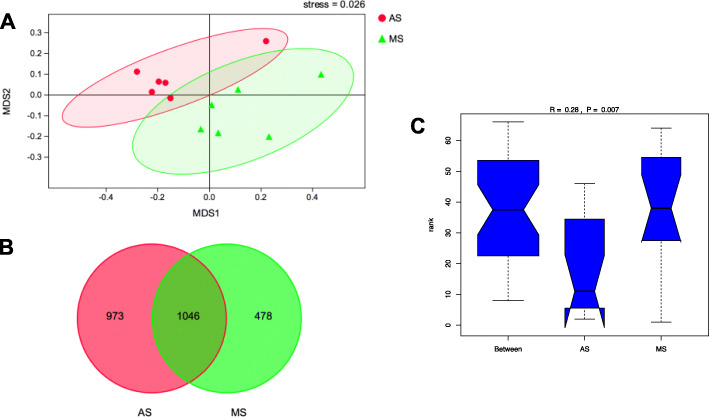
Unweighted non-metric multi-dimensional scaling (NMDS) of taxonomical classifications of bacterial communities (**A**). Venn diagram illustrating overlap of microbial operational taxonomic units (OTUs) between the two groups (**B**). Anosim (analysis of similarities) based on bray-curtis distances between the two groups (**C**). AS, alfalfa silage based diet; MS, mulberry leaf silage based diets

The Good’s coverage of all samples was above 0.99. The Chao, Sobs, Shannon, and Ace indexes of bacterial richness and diversity were different between AS and MS (Table S2). The NMDS plots (Fig. 1a) showed that the clouds derived from the AS and MS data were clearly separated from each other with a significant stress value (0.026). There were 1046 OTUs (operational taxonomic units) that were identified in both of the AS and MS groups, while 973 and 478 specific OTUs were observed in AS and MS groups, respectively (Fig. 1b). The anosim (analysis of similarities) based on bray-curtis distances showed significant different between the two groups (*P* = 0.007).

**Fig. 2:**
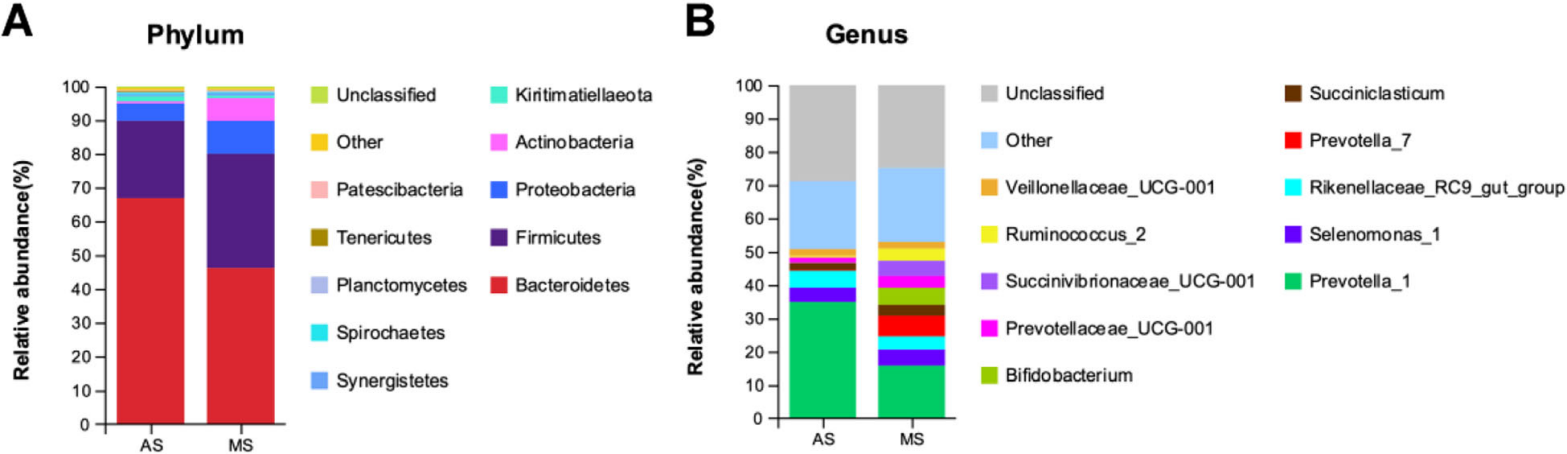
Relative abundance of bacteria community proportions at phylum (**A**) and genus (**B**) level between alfalfa silage (AS) and mulberry leaf silage (MS) treatments (as a percentage of the total sequence)

**Fig. 3:**
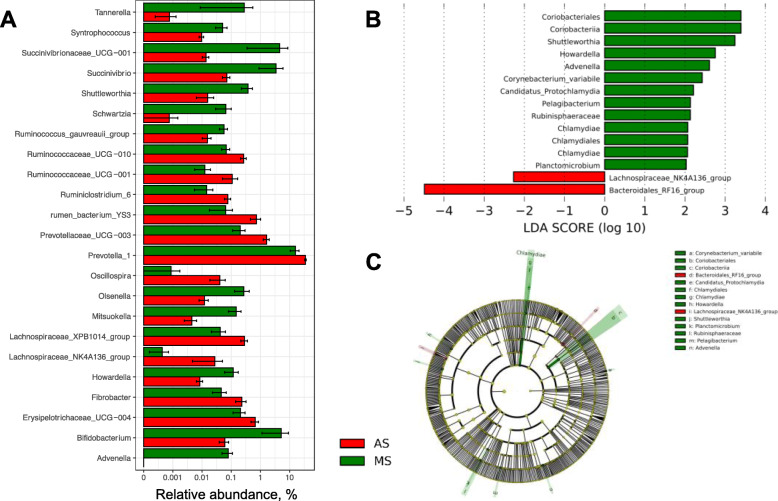
The core specific bacterial biomarker. Significantly different genus bacteria between alfalfa silage (AS) and mulberry leaf silage (MS) treatments (**B**) were tested by Wilcoxon rank-sum test with *P*-value of < 0.05 (**A**). LEfSe (Linear discriminant analysis Effect Size) determined using the Wilcoxon rank sum test (*P* < 0.05) with a linear discriminant analysis (LDA) score analysis shows differentially abundant bacteria communities between alfalfa silage (AS) and mulberry leaf silage (MS) treatments (**B**); The cladogram shows the taxonomic levels represented by rings with six layers from the inside of this plot to the outside, corresponding to six levels of taxonomy (kingdom, phylum, class, order, family, and genus). Each node (small circle) represents a taxon (**C**). Green and red columns or nodes represent the bacteria with the significant higher relative abundance in MS and AS group, respectively

Five bacterial phyla were identified in the rumen samples that had relatively higher abundances (> 1%), including *Bacteroidetes*, *Firmicutes*, *Proteobacteria*, *Actinobacteria*, and *Kiritimatiellaeota*, among them, *Bacteroidetes* was significantly lower in MS group compare with AS group (Fig. 2a). There were 106 bacterial taxa identified at the genus level, and top 10 genera were present with relatively high abundances (Fig. 2b), including *Prevotella_1*, *Selenomonas_1*, *Rikenellaceae_RC9_gut_group*, *Prevotella_7*, *Succiniclasticum*, *Bifidobacterium*, *Prevotellaceae_UCG-001*, *Succinivibrionaceae_UCG-001*, *Ruminococcus_2*, and *Veillonellaceae_UCG-001*. We identified 23 genera as different bacteria based on the Wilcoxon rank-sum test (Fig. 3a), in which, 12 genera were more abundant in the MS samples, including *Advenella*, *Tannerella*, *Succinivibrionaceae_UCG-001*, *Schwartzia*, *Bifidobacterium*, *Succinivibrio*, *Mitsuokella*, *Shuttleworthia*, *Olsenella*, *Howardella*, *Syntrophococcus*, and *Ruminococcus_gauvreauii_group*. and 11 genera were with lower abundance in MS group, such as *Prevotella_1*, *Erysipelotrichaceae_UCG-004*, *Ruminococcaceae_UCG-010*, *Fibrobacter*, *Ruminiclostridium_6*, *Oscillospira*, *Prevotellaceae_UCG-003*, *rumen_bacterium_YS3*, *Ruminococcaceae_UCG-001*, *Lachnospiraceae_XPB1014_group*, and *Lachnospiraceae_NK4A136_group*. As shown in the non-strict version of LEfSe analysis (Fig. 3b, c), 14 clades were more abundant in the MS samples, in which six genera were shown out as *Howardella*, *Pelagibacterium*, *Protochlamydia*, *Shuttleworthia*, *Advenella*, *Planctomicrobium*. Two clades were more abundant in the AS samples, in which the genus *Lachnospiraceae_NK4A136_group* was enriched.

**Fig. 4:**
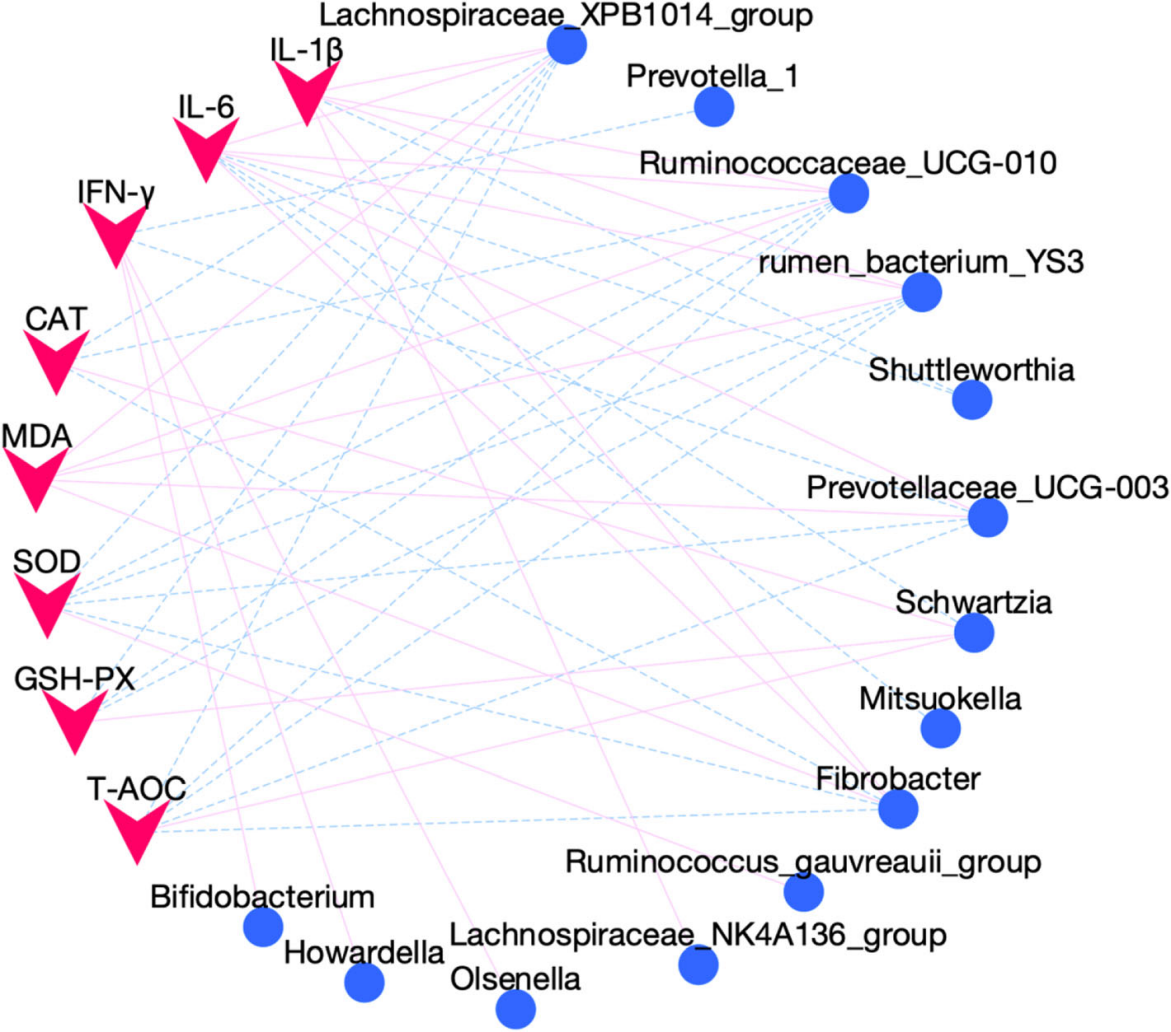
Interactions between rumen different microbiome and serum differential antioxidant and immune parameters. Pearson’s correlations network showing relationships between rumen microbiota and mulberry leaf silage-associated index in serum. Only strong correlations (*r* > 0.59 or r < − 0.59, *P* < 0.05) were showed in the correlation networks. Red straight line, positive correlation (*r* > 0.59); blue dotted line, negative correlation (*r* < − 0.59). Microbes are shown by blue round nodes, and serum parameters are shown by pink V-shaped nodes

It was found that the ruminal bacteria communities were related to the blood antioxidant activity ad immune function indices (Fig. 4). In detail, *Lachnospiraceae_XPB1014_group* and *Ruminococcaceae_UCG-010* were both positively correlated with MDA (*r* = 0.73, *P* < 0.01; *r* = 0.70, *P* < 0.05), but negatively correlated with CAT (*r* = − 0.63, *P* < 0.05; *r* = − 0.66, *P* < 0.05), GSH-PX (*r* = − 0.76, *P* < 0.01; *r* = − 0.64, *P* < 0.05), SOD (*r* = − 0.73, *P* < 0.01; *r* = − 0.71, *P* < 0.05), and T-AOC (*r* = − 0.77, *P* < 0.01; *r* = − 0.70, *P* < 0.05). *Fibrobacter* was positively correlated with MDA (*r* = 0.60, *P* < 0.05), but negatively correlated with SOD (*r* = − 0.61, *P* < 0.05) and T-AOC (*r* = − 0.60, *P* < 0.05). The *rumen_bacterium_YS3* was positively correlated with MDA (*r* = 0.68, *P* < 0.05), but negatively correlated with GSH-PX (*r* = − 0.71, *P* < 0.01), SOD (*r* = − 0.66, *P* < 0.05), and T-AOC (*r* = − 0.69, *P* < 0.05). The *Schwartzia* was positively correlated with GSH-PX (*r* = 0.61, *P* < 0.05), CAT (*r* = 0.61, *P* < 0.05), and T-AOC (*r* = 0.58, *P* < 0.05). *Ruminococcus_gauvreauii_group* was positively correlated with SOD (*r* = 0.59, *P* < 0.05). *Prevotellaceae_UCG-003* was positively correlated with MDA (r = 0.62, *P* < 0.05), but negatively correlated with SOD (*r* = − 0.63, *P* < 0.05), T-AOC (*r* = − 0.62, *P* < 0.05), and IFN-γ (*r* = − 0.64, *P* < 0.05). *Bifidobacterium* (*r* = 0.76, *P* < 0.01), *Howardella* (*r* = 0.72, *P* < 0.01), and *Olsenella* (*r* = 0.62, *P* < 0.05) were positively and *Prevotella_1* (*r* = − 0.75, *P* < 0.01), was negatively correlated with IFN-γ. *Fibrobacter* (*r* = 0.72, *P* < 0.05), *Lachnospiraceae_NK4A136_group* (*r* = 0.61, *P* < 0.05), *Lachnospiraceae_XPB1014_group* (*r* = 0.80, *P* < 0.01), *rumen_bacterium_YS3* (*r* = 0.64, *P* < 0.0*5),* and *Ruminococcaceae_UCG-010* (*r* = 0.80, *P* < 0.01) were positively*, Shuttleworthia* (*r* = − 0.70, *P* < 0.01) was negatively correlated with IL-1β. *Fibrobacter* (*r* = 0.66, *P* < 0.05), *Lachnospiraceae_XPB1014_group* (*r* = 0.78, *P* < 0.01), *Prevotellaceae_UCG-003* (*r* = 0.62, *P* < 0.05), *rumen_bacterium_YS3* (*r* = 0.62, *P* < 0.05), and *Ruminococcaceae_UCG-010* (*r* = 0.76, *P* < 0.01) were positively*, Schwartzia* (*r* = − 0.63, *P* < 0.05), *Shuttleworthia* (*r* = − 0.65, *P* < 0.05), and *Mitsuokella* (*r* = − 0.63, *P* < 0.05) were negatively correlated with IL-6.

## Discussions

The biomass yield of fresh mulberry leaves is approximately 25 to 30 tons/ha/year, and the mulberry leaf is rich in protein (15–35%) [3]. Therefore, mulberry leaves might be used as an excellent protein or feed supplement in animals [3, 17]. In the current study, we found that the animal growth performance was maintained when mulberry leaf silage replaced alfalfa silage, which might be due to their similar protein level and digestible nutrients [18, 19]. Furthermore, we also found that the mulberry leaf silage displays stronger antioxidant activities and immunomodulatory, which has not been reported in sheep. Oxidative stress, induced by reactive oxygen species and free radicals, is usually regarded as one of the causes of cell damage and even disease during nowadays high-intensive farming conditions, which inhibits ruminant growth and induces production loss [20, 21]. Free radicals can be eliminated by antioxidant enzymes such as SOD, GSH-PX, CAT, etc. [22], thus the activities of antioxidant enzymes are a reflection of the antioxidative function of animal. The supplementation of exogenous antioxidants or utilizing plant source rich in antioxidants could be effective to prevent oxidative stress and improve animal health [21, 23]. It was reported mulberry leaves could be a source of natural antioxidants to combat oxidative stress in livestock production due to its scavenging of free radicals in animal feed [7]. Flavonoids and phenolic acids, presenting in mulberry leaf, were the large number of phytochemicals [24]. It was also found that the mulberry leaf is rich polyphenols, such as neochlorogenic acid, chlorogenic acid, rutin, quercetin, astragalin, and kaempferol [25], and the mulberry bioactive compounds showed the antioxidative capacity and anti-inflammatory effects [13, 26]. The anti-oxidant effect of mulberry leaf flavonoids was associated with increased SOD, CAT and GSH-PX expression, as well as reduced MDA levels [27, 28]. It has been also reported that mulberry leaves could benefit animals through the enhancement of serum T-AOC and the activities of CAT and SOD [7]. In the present study, the activity of serum CAT, GPX-PX, SOD, and T-AOC were increased and the serum MDA was reduced after lambs fed with mulberry leaf silage, confirming the improved antioxidant status in the Tan-lambs due to the preserved bioactive components in mulberry leaf silage [13].

The effective of mulberry leaf or its bioactive compounds in improving antioxidant capacity and immune function has been found in many previous studies what we talked above and was confirmed in the current study. To date, there have been few reports on the relationship between the effect roles of mulberry leaf silage on ruminal microbial community and the potential microbial targeted terminal function in antioxidant activity and immunomodulatory. In a previous study, the ruminal microbiota composition of finishing steers was not changed by partial replacement of corn grain and cotton seed meal with ensile mulberry leaves [29]. However, Tan et al. [30] reported that the inclusive of mulberry leaf in diet can not only stimulate the animal growth, but manipulate the ruminal microorganisms in cattle. In the current study, we found several genera bacteria were enhance or decreased by the feeding of mulberry leaf silage, of these changed bacteria, several of them also showed significantly correlated with host serum metabolism. The *Prevotella* accounts to the abundant genera in sheep rumen in the present study, which was also shown in the Hu-sheep [31]. It was also reported that the *Prevotella* genus has the ability in proteolytic [32]. It was found that ruminal *Prevotella* species may affect the amino acids metabolism in dairy cow [16]. We also found the improved serum BUN in MS group. Thus, the nitrogen utilization efficiency might be reduced by decreasing the abundance of *Prevotella_1* in Tan-lambs fed with mulberry leaf silage.

It was also found that the *Schwartzia* was increased in the MS group compared to the AS group. The mulberry leaf flavonoid showed the ability in methane inhibiting in sheep [33]. *Schwartzia* was found to be negatively correlated with methane emissions [34]. Thus, the detected increased abundance of *Schwartzia* in the MS group from the current study and its positively correlated with antioxidant function might be due to the roles of *Schwartzia* in competing with methanogenic bacteria. Thus, these results may suggest the inhibited methane emission when lambs fed with mulberry leaf silage.

The immunoreaction is closely related to health of animals [35]. IFN-γ is involved in the initiation and regulation of the immune response [36]. Cumulative evidences indicate that mulberry leaf polysaccharides have immunomodulating activity, which has been assessed on many different kind cells and macrophage-dependent immune system responses [37]. It was found that water extracts of mulberry leaf mitigate inflammation through the interactions among insulin signalling pathway and TNF-α [38]. However, the current study did not find the changes of serum glucose and TNF-α, but rather with increased serum IFN-γ concentration. Mulberry leaves silage probably contains polyphenols and polysaccharides that could exert similar effects, as polyphenols and polysaccharides can inhibit inflammatory processes by improving serum IFN-γ concentration or affecting microbiota [39, 40]. Here, we show, to our knowledge for the first time, the ruminal *Bifidobacterium* and *Lactobacillus* genera were increased by feeding mulberry leaf silage in sheep which were also positively correlated with the serum IFN-γ concentration in the current study. It was found the bacterial supernatants of the *Bifidobacterium* and *Lactobacillus* genera, are able to affect ghrelin receptor (growth hormone secretagogue receptor-1a) signaling, which plays a crucial role in maintaining energy balance and metabolism, and modulating food intake, motivation, reward, and mood in human being [41]. Thus, in total, we estimated that the improved level of *Bifidobacterium* and *Lactobacillus* genera in rumen of MS group might contribute to the improved healthy level and status of lambs fed with mulberry leaf silage. The increased *Bifidobacterium* in rumen of lamb were associated with the improved contents of meat nutrients comprising alpha-linolenic acid, conjugated linoleic acid and eicosapentaenoic acid [42]. *Lactobacilli* and *Bifidobacterium* are considered by the European Food Safety Authority, indicating they can be considered possible prebiotics for improvement of gut health [43]. *Bifidobacteria* was abundant in the digestive tract of humans, which has assumed health-promoting activities [44]. Vlková et al. [45] also found that the supplementation of *Bifidobacterium* strains promoted rumen health for calves in the milk-feeding period. On the other hand, *Bifidobacteria* can utilize a variety of isoflavones, mono- and oligo- saccharide, and polysaccharides [43]. Mulberry leaf contains abundant polysaccharides and other plant secondary metabolites [40, 46], thus the increased abundance of rumen *Bifidobacteria* in the MS group might due to the feeding of mulberry leaf silage. Our findings for the increased abundance of rumen *Bifidobacteria* in MS group compared to AS group and its highly positively correlation with serum IFN-γ seem to indicate the beneficial effects of genus *Bifidobacterium* on Tan-sheep.

In addition, the IL-1β, a master proinflammatory cytokine, acts as an adjuvant to stimulate antigen-specific immune responses [47]. IL-2 and IL-6 has both pro- and anti-inflammatory activity [48]. Therefore, the much lower IL-1β and IL-6 in MS compared to AS group further confirmed the immunomodulating roles of mulberry leaf in Tan-lambs. In the current study, rumen *Ruminococcac-eae_UCG_010* and *Lachnospiraceae_XPB1014_group* were down-regulated by feeding lambs mulberry leaf silage, which were also negatively correlated with serum antioxidant parameters and positively correlated with serum IL-1β and IL-6. *Ruminococcaceae UCG-010* is reported to be associated with ruminal biohydrogenation [49]. The abundances of *Lachnospiraceae_XPB1014_group* were negatively correlated with body fat weight in finishing pigs, indicating its function in lipid metabolism [50]. Thus, the reduced abundance of *Ruminococcac-eae_UCG_010* and *Lachnospiraceae_XPB1014_group* in MS group indicated the inhibited biohydrogenation in rumen. Thus, the mulberry leaf silage may have the ability to prevent the ruminal biohydrogenation by decreasing *Ruminococcaceae UCG-010* and *Lachnospiraceae_XPB1014_group*, then resulting in more absorption of unsaturated fatty acids that have beneficial roles in antioxidant and immune-enhancing properties [51, 52].

Additionally, except for the described potentially differential functional bacterial, we observed that *Fibrobacter*, *Lachnospiraceae_AC2044_group, Prevotellaceae UCG-003*, and *rumen_bacterium_YS3* were negatively correlated with the enhancement of serum antioxidant characteristics and immune function. In addition, we also found the increased abundance of *Howardella*, *Pelagibacterium*, *Protochlamydia*, *Shuttleworthia*, *Advenella*, and *Planctomicrobium* were achieved in the MS group. However, we did not find the related function of these bacteria. More attention should be paid in detecting their function in further studies by screening and culture methods, as most of their potential function of them in restricting rumen health and host health is still unknown. On the other hand, we have to say that the limitation of this study is that more other immune activity related parameters or cytotoxicity test were not used to evaluated the immune response of animals. In total, our research provides a preliminary view of the efficient utilization of mulberry leaf silage in lambs, and we detect several potential mulberry leaf silage-associated rumen bacteria that may interact with the host metabolism and account for the enhanced antioxidant capacity and immunomodulatory. However, the beneficial roles of these rumen bacteria should be checked and confirmed in further studies.

## Conclusions

We here identify a potent effect of mulberry leaf silage-associated ruminal microbiota interact with host metabolism and physiology. The enriched ruminal *Bifidobacterium*, *Lactobacillus*, and *Schwartzia*, and the down-regulated ruminal *Ruminococcaceae UCG-010* and *Lachnospiraceae_XPB1014_group* were found in the MS group compared to the AS group. Furthermore, these specific changed bacteria that were correlated with the enhanced animal antioxidant capacity or immunomodulatory might be the functional microbes associated with ruminant health. Altogether, evidence from the results of the current study suggest that the naturally mulberry leaf silage could be an alternative functional supplement in maintaining ruminant health and changing the rumen bacterial community. Further experiments should be conducted to verify the function of these rumen bacteria in antioxidant capacity or immunomodulatory.

## Methods

### Silage preparation

The seeds of alfalfa (Beijing Zhengdao Seed Industry Co. LTD, Beijing, China) and mulberry (Suqian Qiuyang Seed Industry Co. LTD, Suqian, China) were obtained commercially. Alfalfa and mulberry were cultivated and harvested on Wuzhong, Ningxia Hui Autonomous Region (37.99°N, 106.20°E). The fresh alfalfa and mulberry leaf were collected and chopped to 2–3 cm using a forage cutter (Lingong Machinery, Shandong, China). Then, both of the alfalfa and mulberry leaf were mixed with 1% glucose and ensiled with 1 × 10^6^ colony forming units (cfu)/g of fresh material *Lactobacillus plantarum* (GenBank accession number: WCFS1) [53]. Both of these two kind silages were fermented well after 60 d ensiling based on the sensory evaluation and pH value measured by a pH meter (PHS-3C, Shanghai Leijun Experimental Instrument Co., Ltd., Shanghai, China) after the bale silage opening. Then, the ingredients were sampled and analyzed. The dry matter (DM) content of the alfalfa silage and mulberry leaf silage were 32.9 and 28.7%, respectively. The concentrations of crude protein (CP), neutral detergent fiber (NDF), acid detergent fiber (ADF), ash, and ether extract (EE) in alfalfa silage were 15.9, 56.6, 37.4, 14.6 and 1.68% of the DM basis, respectively. The concentrations of CP, NDF, ADF, ash, and EE in mulberry leaf silage were 16.2, 63.4, 39.2, 14.3 and 2.87% of the DM basis, respectively.

### Experimental design and sampling

The consent of this experiment had been obtained from the farm owner of Tianyuan Liangzhong Sheep Farm (Ningxia Hui Autonomous Region, China) before the implementation. Forty healthy Tan lambs (*Ovis aries*) with average age of 75 d (± 3), BW of 15.3 kg (± 1.92 SD) were selected from were selected from 5000 sheep fed in Tianyuan Liangzhong Sheep Farm (Ningxia Hui Autonomous Region, China). The sheep were blocked into 20 groups based on the BW and were randomly allocated within the blocks to 1 of 2 treatments (on a DM basis): 1) alfalfa silage based diet (AS; a diet containing 20% alfalfa silage, *n* = 20), 2) mulberry leaf silage based diet (MS; a diet containing 20% mulberry leaf silage, *n* = 20), the other ingredients were identical (Table S1). The animals in each diet group were randomly placed in 4 pens with 5 lambs in each pen. The two groups lambs were both fed with a basal total mixed ration diet. The AS and MS diets contain (DM basis), 14.6 and 14.7% of CP, 31.1 and 32.4% of NDF, 17.8 and 18.2% of ADF, 4.70 and 4.94% of EE, 6.20 and 6.15% of ash, respectively. The feed intake was recorded daily and was measured based on the difference between the amount of feed deliveries and the remaining. The experiment lasted for 80 days, including a 20 days’ adaptation and 60 days’ formal feeding. All the lambs had ad libitum access to feed and water. The animals were excluded if they were used therapeutic during the feeding experiment.

The BW of each lambs were recorded every 20 days. On the day 80 (the end of the experiment), six lambs in each group were then selected for blood sampling and slaughtering based on the average BW. Blood was collected from the jugular vein of the sheep to separate serum (centrifuged for 10 min at 3000×g). All the lambs were sacrificed in a public abattoir using carotid bleeding on day 80 at 10:00 am (3 h after morning feeding). After slaughtering, the weight of carcass, head, skin, limbs, internal organs (heart, liver, spleen, lung, kidney) were measured and recorded. All the collected samples were then stored at liquid nitrogen for subsequent analysis.

The detail information of the meat quality detection was according to a previous study of Liang et al. [54]. In brief, body fat (assessed as GR) value was assessed by measuring the thickness at the 12th/13th rib intersection 110 mm away from the midline using a vernier caliper. The pH value and meat colour (redness (a*), yellowness (b*), and lightness (L*), psychometric chroma (c* = (a^2^ + b^2^)^0.5^), and Hue angle (H* = arctan (b*/a*) × (180/π)) at 45 min and 24 h were measured. Meat colour of longissimus thoracis was measured using a high-quality spectrophotometer NS800 (3NH technology Co. LTD, Shenzhen, China) with illuminant D65 and a viewing angle of 10 °. Each sample was determined three times at different positions on the meat surface and the average value was obtained. Serum metabolite concentrations, including total protein, albumin, BUN, triacylglycerol, total cholesterol, high density lipoprotein cholesterol, low density lipoprotein cholesterol, immunoglobin G, immunoglobin A, and immunoglobin M were measured using an automatic analyzer (Kehua ZY KHB-1280, Shanghai, China) with a commercial kits (Shanghai Kehua Biological Technology Co. Ltd., Shanghai, China) [55]. The activities of antioxidant enzymes such as SOD, CAT, GSH-PX, T-AOC and MDA, and the concentrations of IFN-γ, TNF-α, IL1-β, IL-2, and IL-6 were determined by commercial kits (Jiancheng Biological Technology Co. Ltd., Nanjing, China) following the manufacturer’s instructions.

Rumen fluid (20 mL) was collected from the ventral part of the rumen by straining the ruminal content through four layers of cheesecloth. Rumen fluid pH was measured immediately. Rumen fluid samples were then stored at liquid nitrogen for subsequent analysis. The concentrations of volatile fatty acids were measured by gas chromatography (Trace 1300; Thermo Fisher Scientific Co., Ltd., Shanghai, China). The ammonia nitrogen was determined by the method described in Broderick and Kang [56].

### Profiling of rumen bacterial community diversity

Microbial DNA from rumen fluid was extracted by a HiPure Stool DNA Kit (Magen, Guangzhou, China) according to manufacturer’s protocols. The 16S rDNA V3-V4 region of the ribosomal RNA gene were amplified by PCR using primers 341F: CCTACGGGNGGCWGCAG; 806R: GGACTACHVGGGTATCTAAT following the procedure described by Sun et al. [57]. The amplicons were purified by the AxyPrep DNA Gel Extraction Kit (Axygen Biosciences, Union City, CA, U.S.). Then it was quantified by ABI StepOnePlus Real-Time PCR System (Life Technologies, Foster City, USA).

Purified amplicons were pooled in equimolar and paired-end sequenced (PE250) on an Illumina platform (Illumina Novaseq 6000 sequencing) according to the standard protocols. Raw reads were further filtered using FASTP to get high quality clean reads [58]. The noisy sequences of raw tags were filtered by QIIME (version 1.9.1) [59]. Then, chimeric tags were removed using UCHIME algorithm. The finally obtained effective tags were clustered into OTUs of ≥97% similarity. Based on the SILVA database (version 132), the representative sequences were assigned to organisms by a naive Bayesian model using RDP classifier (Version 2.2). The abundance statistics of each taxonomy were constructed using a Perl script and visualized by SVG. The alpha index including Chao, Simpson, Sobs, Shannon, and Ace were calculated in QIIME. Unweighted non-metric multi-dimensional scaling (NMDS) was generated in R project Vegan package (version 2.5.3). Statistical analysis of Wilcoxon rank test and Anosim test was calculated in R project Vegan package (version 2.5.3). The bacterial community comparison between the AS and MS groups was calculated by Wilcoxon rank test in R project Vegan package (version 2.5.3). Pearson correlation coefficient between environmental factors and species and between blood parameters and species was calculated in R project psych package (version 1.8.4). Network of these correlation coefficients were generated using igraph package (version 1.1.2) in R project.

### Statistical analyses

The animal performance, blood parameters, and rumen fermentation characteristics data were analyzed for a completely random design using the PROC MIXED procedure of SAS (version 9.4, SAS Institute Inc., Cary, NC). The means of each treatments are presented as least squares means and statistical significance was defined at *P* < 0.05, and the trends were declared at 0.05 ≤ *P* ≤ 0.10.

## Supplementary Information

**Additional file 1: Table S1.** Ingredients and nutrient composition of the total mixed ration containing alfalfa silage (AS) and mulberry leaf silage (MS) as the main forage. **Table S2.** Alpha diversity indices of rumen bacteria in lambs fed alfalfa silage (AS) and mulberry leaf silage (MS) based diets.

## Data Availability

The data sets generated during and/or analyzed during the current study are available from the corresponding author on reasonable request. The raw reads of 16S rRNA sequencing data have been deposited into the NCBI Sequence Read Archive (SRA) database (Accession Number: SRP265735).

## Abbreviations

ADG: Average daily gain
ADF: Acid detergent fiber
AS: Alfalfa silage diet
BUN: Blood urea nitrogen
CAT: Catalase
CP: Crude protein
DM: Dry matter
EE: Ether extract
GSH-PX: Glutathione peroxidase
MDA: Malondialdehyde
MS: Mulberry leaf silage diet
NDF: Neutral detergent fiber
SOD: Superoxide dismutase
T-AOC: Total antioxidant capacity
