# Supplementary material for: Effects of mulberry leaf silage on antioxidant and immunomodulatory activity and rumen bacterial community of lambs
Source: BMC Microbiol. 2021 Sep 20;21:250. doi: 10.1186/s12866-021-02311-1 (PMC8454139; doi:10.1186/s12866-021-02311-1)
Supplement: Supplementary file 1 — Additional file 1: Table S1. Ingredients and nutrient composition of the total mixed ration containing alfalfa silage (AS) and mulberry leaf silage (MS) as the main forage. Table S2. Alpha diversity indices of rumen bacteria in lambs fed alfalfa silage (AS) and mulberry leaf silage (MS) based diets. [file 12866_2021_2311_MOESM1_ESM.docx]

***Supplementary Material***

**Effects of mulberry leaf silage on antioxidant and immunomodulatory activity and rumen bacterial community of lambs**

**Bing Wang and Hailing Luo***

*State Key Laboratory of Animal Nutrition, College of Animal Science and Technology, China Agricultural University, Beijing 100193, P. R. China*

*Corresponding Author:

Prof. Hailing Luo

*Email address:* luohailing@cau.edu.cn

**Additional file 1**

**Table S1**

Ingredients and nutrient composition of the total mixed ration containing alfalfa silage (AS) and mulberry leaf silage (MS) as the main forage.

| Items, dry matter basis | Treatments | |
| --- | --- | --- |
|  | AS | MS |
| Ingredients, % |  |  |
| Corn grain | 38.7 | 38.7 |
| Wheat bran | 11.8 | 11.8 |
| Soybean meal | 10.2 | 10.2 |
| Alfalfa hay | 10.0 | 10.0 |
| Corn silage | 0.0 | 0.0 |
| Alfalfa silage | 20.0 | 0.0 |
| Corn stover | 5.0 | 5.0 |
| Mulberry leaf silage | 0.0 | 20.0 |
| NaHCO3 | 0.6 | 0.6 |
| Premix^1^ | 3.7 | 3.7 |
| Chemical composition |  |  |
| Crude protein, % | 14.6 | 14.7 |
| Neutral detergent fiber, % | 31.1 | 32.4 |
| Acid detergent fiber, % | 17.8 | 18.2 |
| Non-fiber carbohydrate, % | 43.4 | 41.8 |
| Ether extract, % | 4.70 | 4.94 |
| Ash, % | 6.20 | 6.15 |
| Ca, % | 0.84 | 0.98 |
| P, % | 0.44 | 0.43 |
| Metabolic energy, MJ/kg | 10.0 | 10.1 |

^1^Formulated to provide (per kilogram of dry matter): 500,000 IU of vitamin A, 160,000 IU of vitamin D3, 650 IU of vitamin E, 150 g of NaCl, 20 g of Ca, 20 g of P, 1750 mg of Zn, 15 mg of Se, 50 mg of I, 2000 mg of Fe, 20 mg of Co, 1500 mg of Mn, and 600 mg of Cu.

**Table S2**

Alpha diversity indices of rumen bacteria in lambs fed alfalfa silage (AS) and mulberry leaf silage (MS) based diets.

| Item | Sobs | Shannon | Simpson | Chao | Ace |
| --- | --- | --- | --- | --- | --- |
| AS | 1908 | 7.22 | 0.97 | 2406 | 2494 |
| MS | 1542 | 6.31 | 0.95 | 2045 | 2141 |
| SEM | 120.6 | 0.337 | 0.012 | 140.9 | 140.6 |
| *P*-value | 0.04 | 0.04 | 0.59 | 0.04 | 0.04 |
